# Effects of Body Condition and Ectoparasitism on Host–Pathogen Interactions of Heteromyid Rodents

**DOI:** 10.3390/pathogens13121085

**Published:** 2024-12-09

**Authors:** Diana M. Beristain-Ruiz, Ana K. Márquez-Chacón, Cuauhcihuatl Vital-García, Julio V. Figueroa-Millán, José J. Lira-Amaya, John F. Aristizabal, Martha P. Olivas-Sánchez, Ana B. Gatica-Colima, Jesús M. Martínez-Calderas, Andrés Quezada-Casasola, Beatriz Alvarado-Robles, Víctor M. Alonso-Mendoza

**Affiliations:** 1 Departamento de Ciencias Veterinarias, Universidad Autónoma de Ciudad Juárez, Anillo Envolvente y Estocolmo s/n Colonia Progresista AP 1729-D Cd. Juárez, Chihuahua CP 32310, Mexico; diana.beristain@uacj.mx (D.M.B.-R.); al229144@alumnos.uacj.mx (A.K.M.-C.); aquezada@uacj.mx (A.Q.-C.); balvarad@uacj.mx (B.A.-R.); valonso@uacj.mx (V.M.A.-M.); 2 CENID-Salud Animal e Inocuidad, Instituto Nacional de Investigaciones Forestales, Agrícolas y Pecuarias, Cuernavaca-Cuautla 8534 Colonia Progreso, Jiutepec, Morelos CP 62574, Mexico; figueroa.julio@inifap.gob.mx (J.V.F.-M.); lira.juan@inifap.gob.mx (J.J.L.-A.); 3 Departamento de Ciencias Químico-Biológicas, Instituto de Ciencias Biomédicas, Universidad Autónoma de Ciudad Juárez, Av. Plutarco Elías Calles 1210, Fovissste Chamizal, Cd. Juárez, Chihuahua CP 32310, Mexico; john.aristizabal@uacj.mx (J.F.A.); polivas@uacj.mx (M.P.O.-S.); agatica@uacj.mx (A.B.G.-C.); jesus.calderas@uacj.mx (J.M.M.-C.)

**Keywords:** *Rhipicephalus sanguineus*, *Meringis* spp., *Yersinia pestis*, *Rickettsia* spp., heteromyid rodents

## Abstract

Rodents play a significant role in the transmission of zoonotic diseases; anthropization has increased human contact with these animals, vectors of infectious agents. However, the processes driving parasitism of hosts remains poorly understood. *Yersinia pestis*, *Rickettsia* spp., and *Francisella tularensis* are three infectious agents transmitted to humans through ectoparasites, with rodents serving as the primary reservoirs. To explore the relationship between both intrinsic and extrinsic factors on host pathogen status, we evaluated heteromyid rodents in the Chihuahuan desert (ChD). From December 2022 to May 2023, we sampled 213 rodents at three locations with different anthropization levels. A total of 103 rodent blood samples, 84 organ samples, and 204 collected ectoparasites were analyzed for molecular detection of infectious agents (*Y. pestis*, *Rickettsia* spp., and *F. tularensis*) with PCR. We captured seven species of rodents (*Dipodomys ordii*, *D. merriami*, *D. spectabilis*, *Chaetodipus hispidus*, *Ch. eremicus*, *Perognathus flavus*, and *P. flavescens*) and identified one tick (*Rhipicephalus sanguineus*), two fleas (*Meringis altipecten* and *M. dipodomys*) and one louse (*Fahrenholzia* spp.). Molecular analyses yielded positive for *Y. pestis*, *Rickettsia* spp., and negative for *F. tularensis*. We then modelled the pathogen status as a function of intrinsic (body condition and sex) and extrinsic factors (locality, anthropization level, season, sample type, and parasite-infestation status). We found that non-parasite-infested individuals with better body condition have a higher probability of pathogen infection. Furthermore, we observed that blood samples had a higher probability of detecting pathogen-infected individuals, as compared to spleen or liver samples. Our results offer important insights into host–pathogen interactions and the role of body condition in the pathogen status.

## 1. Introduction

Throughout history, rodents have played a significant role in the transmission of zoonotic diseases and are considered to be of great importance to public health [1]. Many rodent species have been proposed as potential reservoirs of new zoonotic diseases, with some species classified as hyperreservoirs, carrying between two and eleven zoonoses [2]. Furthermore, rodents are distributed worldwide, representing over 40% of mammalian global biodiversity [3], and they are well adapted to sharing habitats with humans. Recent changes in both climate and urbanization have facilitated the range expansion of some rodent species, as many human activities create conditions that are suitable for them, providing food and shelter [4], thereby increasing rodent abundance [5,6] and potential contact with humans. Nevertheless, the parasitism load within populations is highly variable; while some individuals carry many parasites, others carry few [7].

The processes driving parasitism of hosts are not fully understood; the presence of parasites or parasitism depends on individual risks factors, such as sex [8], age [9], body mass, and body condition [10], and these have been proposed to be crucial in determining parasite infestation and interindividual variation [11]. Body condition is used as a proxy of animal health and has been used to infer the effects of parasitism and pathogen infection over the host [12]. Nevertheless, better body condition has been suggested to increase parasitism loads [13,14].

In addition to intrinsic factors, extrinsic factors may also contribute to the heterogeneity of parasite infestation [15]. Land use change, for instance, favors an abundance and diversity of reservoir hosts, affecting host–pathogen dynamics [6,16]. Parasite dynamics may also be influenced by climatic factors, specifically precipitation and temperature [10,17]. Season and humidity [18] influence ectoparasite survival, facilitating ectoparasite development [19].

In this study, we first report the pathogens identified in heteromyid rodents and their ectoparasites in three locations of the Chihuahuan desert and determine whether prevalence is related to ectoparasite–host interaction. We then model pathogen status as a function of host traits (body condition, and sex), and extrinsic factors (locality, anthropization level, season, sample type, and parasite infestation status).

## 2. Materials and Methods

### 2.1. Ethical Approval and Informed Consent

This study received approval and the corresponding permit from SEMARNAT (SPARN/DGVS/02393/22) and was approved by the Ethical and Bioethical Committee of the Autonomous University of Juárez (CEI-2022-2-771), Mexico.

Molecular assessment was conducted at the Laboratory of Veterinary Clinical Pathology and Molecular Biology at the Autonomous University of Juárez and at the Babesia Research Unit within the National Center for Disciplinary Research in Animal Health and Safety (CENID-SAI) of the National Institute of Forestry, Agricultural, and Livestock Research (INIFAP), Mexico.

### 2.2. Study Area and Samples

**Figure 1:**
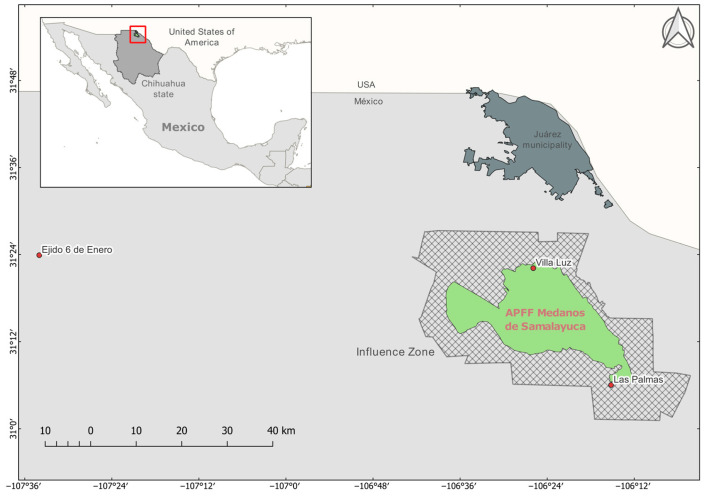
Study site and localities of rodent and parasite collection.

For the present research, three different areas located in the north of the state of Chihuahua were studied (Figure 1):

(a) Ejido Villa Luz is situated in the northern region of APFFMS. Within the ejido, various anthropogenic activities occur, including agriculture, livestock grazing, and even tourism. This area is part of the municipality of Juárez, Chihuahua, and comprises 619 households with a population of 1577 residents [20].
(b) Rancho Las Palmas is within the influence area of the APFFMS, as it is situated adjacent to the southeastern part of the protected area. This ranch, dedicated primarily to cattle, is privately owned, with restricted human mobility, and there are only two houses in the area [20].
(c) Finally, Ejido Ley Seis de Enero is situated in the northern part of the state of Chihuahua, 125 km west of APFFMS. It falls within the municipality of Ascensión, Chihuahua, and comprises 297 households with a population of 671 residents [20].

### 2.3. Trapping and Sampling

**Figure 2:**
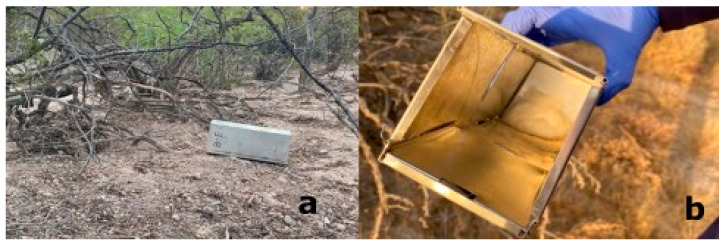
Sherman traps. (**a**) Sherman trap set near a rodent burrow. (**b**) Sherman trap baited with rolled oats.

To capture rodents, 80 Sherman traps (H. B. Sherman Traps, Inc., Tallahassee, FL, USA) were set at 10 m intervals (Figure 2a); each trap had an alphanumeric code and was baited with oat flakes (Figure 2b). Traps were set in the late afternoon, at around 6:00 p.m., near the entrances to rodent burrows and along known rodent trails and inspected the following morning at 6:00 a.m. Sampling was conducted during December–January 2022 and May–June 2023.

Following rodent capture, species were identified using field guides [21]. Standard data for each animal, including weight, morphometric measurements, and notable observations about the rodent’s physical condition, were recorded. Each rodent was then systematically examined for ectoparasites. Ticks were removed using entomological tweezers, while fleas were collected with a toothbrush, which was used to brush the rodent and capture the fleas trapped in its fur. Blood samples were collected intracardially using an insulin syringe. Prior to sampling, each rodent was administered an intraperitoneal dose of xylazine, adjusted according to its weight, as a muscle relaxant and analgesic [22]. Blood samples were then placed in EDTA-coated vacutainer tubes and kept refrigerated at 4 °C until they arrived at the laboratory; they were then stored at −20 °C until molecular analysis was performed. Both ectoparasites and blood samples were labeled with an identification code for tracking purposes.

### 2.4. Rodent Euthanasia and Necropsy

For euthanasia, rodents were first weighed and then anesthetized with an intraperitoneal injection of a mixture of ketamine (75 mg/kg) and xylazine (10 mg/kg). Once fully anesthetized, sodium pentobarbital (175 mg/kg) was administered intraperitoneally [22]. Bodies were transported in plastic bags within a cooler. In the laboratory, necropsies were conducted to extract the liver and spleen, which were then used for laboratory tests to detect pathogens [23].

### 2.5. Ectoparasite Identification

Ectoparasite identification was performed after DNA extraction from the specimens using an optical microscope (Primo Star Iled, ZEISS, Shangai, China). Additionally, microscope images were captured with an Axio Imager A2 (ZEISS, Göttingen, Germany). The identification of ectoparasite structures followed the taxonomic guidelines [24,25,26,27].

### 2.6. DNA Extraction from Ectoparasites and Rodent Blood and Organ Samples

Rodent blood, organ samples, and collected ectoparasites were processed to detect the presence of three pathogens (*Yersinia pestis*, *Rickettsia* spp., and *Francisella tularensis*) by DNA amplification, using endpoint PCR. A commercial kit was used for DNA extraction (DNeasy Blood and Tissue, Qiagen, Hilden, Germany), following the manufacturer’s instructions. However, due to the small size of the study subjects, for blood samples, some modifications were occasionally required to achieve the maximum concentration of DNA (the modifications involved extending the incubation time to 15 min at 56 °C in a dry bath in accordance with the manufacturer’s recommendations). In the case of ectoparasites, several modifications suggested by other authors were implemented to optimize DNA extraction [28]. Finally, for DNA extraction from rodent organs, the manufacturer’s instructions were followed.

We used DNA pooling for the detection of pathogens in ectoparasites. Pools were arranged with all ectoparasites—separated by ticks, fleas, and lice—collected from a single individual. A total of 75 ectoparasite pool samples were analyzed for each pathogen, resulting in 225 PCR tests being performed.

### 2.7. PCR Amplification and Sequencing

For the DNA amplification of blood, organ, and ectoparasite pools, the following reaction mixture was prepared: 5 μL of DNA sample, 1 μL each of forward and reverse primers, 12.5 μL of Green Master Mix (GoTaq, Promega, Madison WI, USA), and 5 μL of nuclease-free water, making up a total volume of 25 μL. A negative control, in which DNA was substituted with nuclease-free water, and a positive control specific to each pathogen (Blue Heron Biotech, Eurofins Genomics, Bothell, WA, USA) were included in each reaction. Once the mixtures were prepared, they were transferred to a thermal cycler (C1000 Touch, Bio-Rad Laboratories Inc., Hercules, CA, USA) to execute the amplification protocol for each primer (Table 1).

The PCR was performed using a C1000 Touch™ Thermal Cycler (Bio-Rad Laboratories, Hercules, CA, USA). The PCR products were then analyzed through agarose gel electrophoresis, stained with 1% ethidium bromide, and examined using a gel documentation system (BioDoc-it 220; UVP Imaging System, Upland, CA, USA).

The PCR products amplified from rodent blood or tick, flea, and lice pools were purified using the Wizard^®^ SV Gel and PCR Clean-Up System (No. Cat A9281, Madison, WI, USA). To validate the identity of the molecularly detected pathogens, three randomly selected positive samples from each pathogen were purified and sent to Eurofins Genomics Laboratory (Louisville, KY, USA) for bi-directional sequencing. The obtained sequences were edited using the MEGA software v. 11 and subsequently aligned with all other sequences from the GenBank database in nBLAST to obtain the percentage of identity; a sequence identity of 98% was considered for correct identification for *Rickettsia* spp. [29], while the *pla* gene sequence in *Y. pestis* is unique and not present in other *Yersinia* species [30].

**Table 1:** Sequences of primer sets and protocols used for PCR detection.

Pathogen	Oligonucleotide Sequence (5′-3′)	PCR Protocol	Reference
*Rickettsia* spp.	gltAF: GCAAGTATCGGTGAGGATGTAAT gltAR: GCTTCCTTAAAATTCAATAAATCAGGAT	Initial denaturation: 95 °C for 5 min, followed by 35 cycles, each consisting of: 95 °C for 30 s, 58 °C for 30 s, 65 °C for 45 s, and final extension at 72 °C for 7 min.	[31]
*Yersinia pestis* (*pla*)	Yp1: ATCTTACTTTCCGTGAGAAG Yp2: CTTGGATGTTGAGCTTCCTA	Initial denaturation 95 °C for 5 min, followed by 35 cycles, each consisting of 95 °C for 1 min, 56 °C for 1 min, 72 °C for 1 min, and final extension at 72 °C for 10 min.	[30]
*Francisella tularensis* (*fopA*)	FNA7L-F: CTTGAGTCTTATGTTTCGGCATGTGAATAG FNB1L-R: CCAACTAATTGGTTGTACTGTACAGCGAAG	Initial denaturation 94 °C for 5 min, followed by 20 cycles, each consisting of 95 °C for 10 s, 62 °C for 10 s, 72 °C for 10 s, and final extension at 72 °C for 5 min.	[32]

### 2.8. Data Analysis and Ectoparasite and Rodent Statistical Model

To explore the effects of body condition, species, sex, locality, anthropization level, season, sample type, and parasite infestation status (infested vs. non infested) over pathogen infection status (infected vs. non-infected), we used generalized linear models (GLMs) fitted with a binary logit distribution using the AIC for model selection. We calculated the body condition index, a scaled mass index, as a function of body length and body mass [33]. We started with a global model with all factors including pathogens identified in sampled rodents. For the selected models, we calculated the relationship between variables by extracting the *p*-value and the slope of the GLM test. We further graphed the explanatory variables related to pathogen infection status with Cleveland diagrams. To estimate the predictive power (sensibility and specificity), we used the receiver operating characteristic (ROC) curve [34]. We used the area under the ROC curve (AUC) as a summary of the predictive value of the model [35] and assumed a minimum value of 0.80 to measure a good discriminative capacity [36]. The GLM was performed using the ‘stats’ package of the statistical program R (version 4.2.2) (R Core Team, 2022). The Cleveland plot was constructed using the ‘dotchart3′ function of the ‘Hmisc’ package [37]. The ROC curve was calculated using the ‘pROC’ package [38].

To assess the prevalence of ectoparasites in heteromyids, we fitted a Beta regression model using ectoparasites prevalence as a dependent variable (continuous variable bounded between 0 and 1). Dependent variables include the type of ectoparasite (*R. sanguineus*, *M. altipecten*, and *Fahrehnholzia* spp.) and rodent species (*D. merriami*, *D. ordii*, *D. spectabilis*, *C. hispidus*, *C. eremicus*, *P. flavus*, and *P. flavescens*). The Beta regression model is appropriate for proportion data that do not include values at the boundaries [39]. Additionally, it is effective for capturing the variability in prevalence data, where the variance is expected to change as a function of the mean [40]. To assess the model’s assumptions, the residuals were examined for their distribution and normality [41]. The autocorrelation of the residuals was evaluated using an autocorrelation function (ACF) plot, where it is expected that the residuals show no significant dependence [41]. Finally, the model fit was assessed using pseudo-R², which represents the percentage of variability explained by the model (values >0.4 are considered a good fit) [40], and the phi parameter (values >1 indicate a good fit), which reflects the accuracy of the adjusted model [39]. A high phi value and a satisfactory pseudo-R^2^ suggest that the model effectively captures the variability in ectoparasite prevalence across rodent species. To visualize the adjusted means of ectoparasite prevalence for each combination of rodent species and ectoparasite type, the emmeans [42] and ggplot2 [43] packages were used. The emmeans package allows for the calculation of adjusted marginal means for the dependent variable (Prevalence), considering the effects of the model’s factors (ectoparasites and rodents) while controlling for other model terms [43]. These adjusted means represent the expected prevalence at each level of the factors, averaged across the levels of the other factors in the model. To adjust the Beta model, the betareg package [44] from the R statistical software (version 4.2.2) (R Core Team, 2024) was used.

## 3. Results

### 3.1. Host Rodent Species

A total of 213 rodents were captured: 90 rodents in winter and 123 in summer (Table 2). The most abundant species was *Dipodomys merriami* (49.76%, n = 106), followed by *Chaetodipus hispidus* (22.06%, n = 47).

### 3.2. Presence and Morphological Identification of Ectoparasites Found in Rodents

During this study, the presence of ectoparasites was identified in the following species: *Dipodomys merriami*, *D. ordii*, *D. spectabilis*, *Chaetodipus hispidus*, *C. eremicus* and *Perognathus flavus*; 89 individuals (42% of the total captures) were parasitized with the presence of at least 1 ectoparasite (Table 3). None of the captured individuals of the species *P. flavescens* were infested. The species most affected by the presence of ectoparasites was *C. hispidus* with a prevalence of 48.9% (23/47).

**Figure 3:**
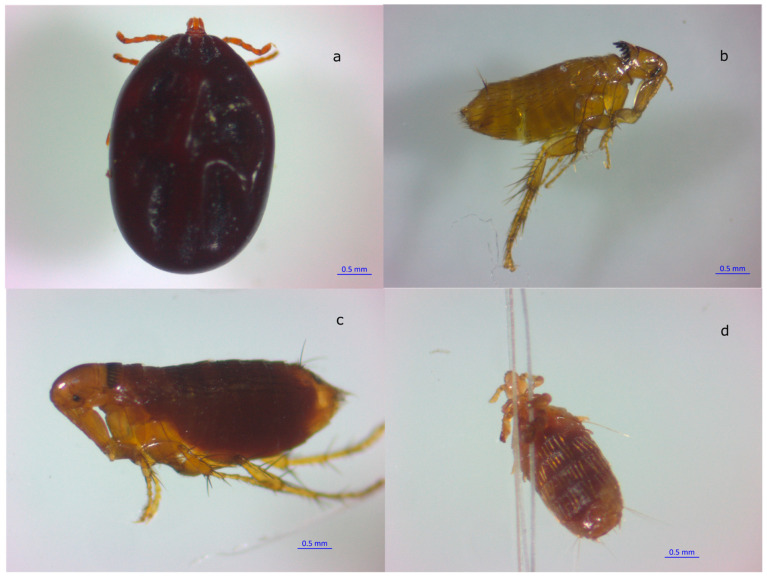
Ectoparasites collected from heteromyid rodents. (**a**) *Rhipicephalus sanguineus*, (**b**) *Meringis altipecten*, (**c**) *Meringis dipodomys*, (**d**) *Fahrenholzia* spp. Blue scale bar: 0.5 mm.

The most abundant ectoparasites found were ticks, representing 52.72% (134/254), followed by fleas at 45.66% (116/254) and lice with a much lower prevalence at 1.57% (4/254). The only tick species identified was *R. sanguineus*, while two flea species were found: *Meringis altipecten*, representing 62.93% (73/116); and *Meringis dipodomys*, representing 37.06% (43/116). Finally, the only species of lice corresponded to *Fahrenholzia* spp. Regarding prevalence, *M. altipecten* and *R. sanguineus* were the most prevalent (Table 3), followed by *M. dipodomys* and finally the louse *Fahrenholzia* spp. *Dipodomys ordii* had the highest prevalence with *M. altipecten*, and *C. hispidus* with *R. sanguineus*. Figure 3 shows some of the ectoparasites collected from the rodents studied.

### 3.3. Prevalence of Tick-Borne Pathogens in Rodents

Regarding blood molecular detection of pathogens, a prevalence of 4.85% (n = 6) was found for *Y. pestis* (four *D. merriami* and one *P. flavescens*), while for *Rickettsia* spp., the prevalence was 3.88% (n = 4, three specimens *Ch. hispidus* and one *D. merriami*). Finally, *F. tullarensis* was not detected in blood samples.

On the other hand, a total of 84 samples (42 liver and 42 spleen) were analyzed for the detection of the three pathogens. Four positive samples for *Y. pestis* were obtained, all of them in the spleens of two *D. merriami* and two more from *C. eremicus*. No positive results were obtained for the other pathogens or any of the liver samples tested. For the detection of *Rickettsia* spp and *F. tullarensis*, no positive results were obtained in the organs analyzed.

### 3.4. Prevalence of Tick-Borne Pathogens on Ectoparasites

Out of the 75 pools analyzed, only positive results were observed for *Rickettsia* spp. in one flea pool, *M. altipecten*, and one tick pool *R. sanguineus*, both pool parasites collected from *D. merriami*.

### 3.5. Intrinsic and Extrinsic Factors Related to Pathogen Infection

**Figure 4:**
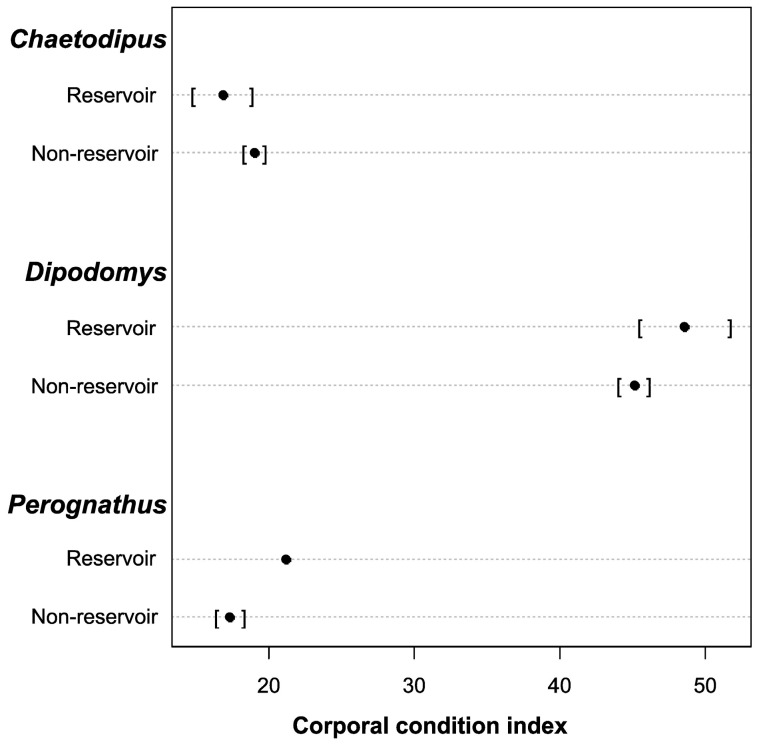
Cleveland diagram of reservoirs vs. non-reservoirs and their relationship with body condition classified by genus. Black dots indicate the mean and standard deviation (in brackets).

**Figure 5:**
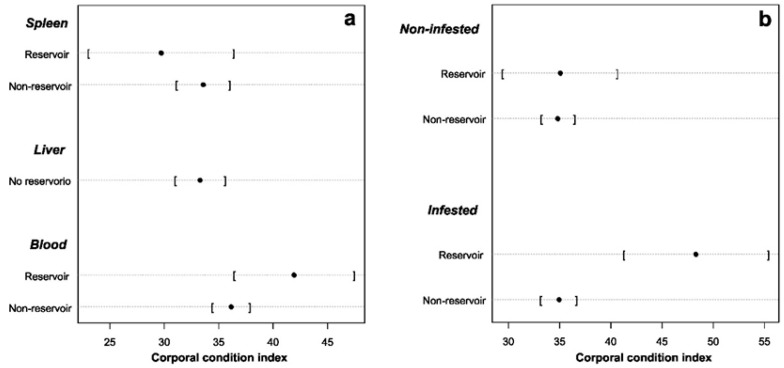
Cleveland diagram of (**a**) reservoirs vs. non-reservoirs in relation to sample origin (liver, spleen and blood) and (**b**) reservoirs vs. non-reservoirs in relation to parasite infestation. Black dots indicate the mean and standard deviation (in brackets).

When considering the effect of body condition, sex, locality, anthropization level, season, sample type, and parasite infestation status over pathogen status (reservoir vs. non-reservoir), we found an interesting pattern. First, we found (Figure 4) that body condition means in *Perognathus* and *Dipodomys* rodents are numerically higher in pathogen-infected rodents than non-infected rodents for these genera. However, *Chaetodipus* does not show a difference in body condition among infected vs. non-infected rodents. Furthermore, we observed better body condition for pathogen-infected individuals who presented parasite infestation (Figure 5b).

Our results suggest that non-infested individuals with better body condition (Figure 5b) have a higher probability of pathogen infection (Table 4). Furthermore, we observed that blood samples had a higher probability of detecting pathogen-infected individuals. We found that blood samples had a 17-fold greater probability of detecting pathogen infection as compared to liver samples (Table 4).

### 3.6. Ectoparasite and Rodent Statistitcal Model

**Figure 6:**
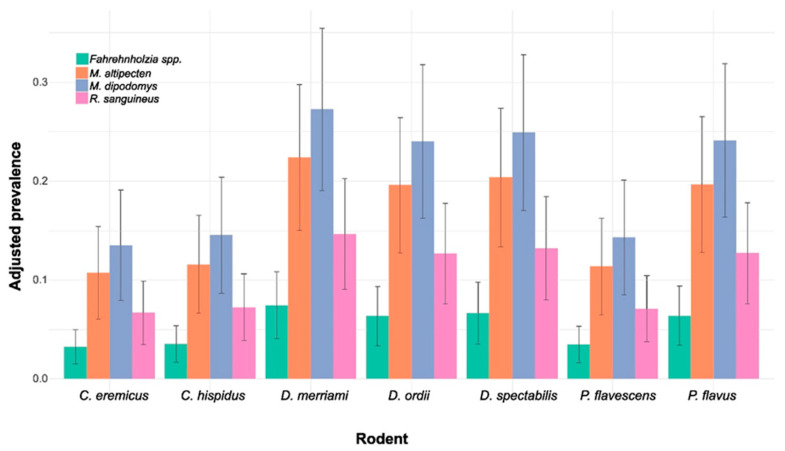
Adjusted means of ectoparasite prevalence by rodent and ectoparasite species. Each bar represents the adjusted mean prevalence for a specific combination of ectoparasite and rodent levels, with error bars indicating the standard error of the estimate.

Our model results (Table 5) indicate that the ectoparasites M. altipecten and *M. dipodomys* have statistically significant coefficients (*p* < 0.01), meaning that they are associated with a higher prevalence compared to the reference category (intercept). In contrast, *R. sanguineus* and *Fahrehnholzia* spp. do not have significant coefficients, suggesting that these ectoparasites do not have a notable effect on the adjusted prevalence relative to other ectoparasites. None of the coefficients associated with the different rodent species are significant, suggesting that, after adjusting for ectoparasite type, rodent species do not have a meaningful effect on prevalence. The prevalence of ectoparasites in rodent species appears to be more strongly influenced by ectoparasite type than by rodent species. This is supported both through graphical representation (Figure 6) and the model results (Table 5), where *M. dipodomys* and *M. altipecten* exhibit a significantly higher prevalence.

The above suggests that both *M. altipecten* and *M. dipodomys* may be adapted to infect a broad range of heteromyid rodents, while other ectoparasites, such as *Fahrehnholzia* spp., exhibit lower overall prevalence. The lack of significance associated with rodent species indicates that these ectoparasites are not specific to any single species, suggesting a broader ecological adaptation of ectoparasites within rodent communities (Figure 6).

## 4. Discussion

Studies around the world indicate that heteromyids are natural reservoirs for different species of ectoparasites [45,46], including Mexico. The ectoparasites reported here correspond to those previously described by other authors [47] and previous studies by our team [14], as they describe various species of fleas found in regions adjacent to our study area, as well as in the same species of rodents. We further report *Y. pestis* and *Rickettsia* spp. identified in heteromyids and their associated parasites as well as the effect of body condition and infestation status on pathogen infection probability; we report no effect of other intrinsic or extrinsic factors on either parasite or pathogen infection status.

*Rhipicephalus sanguineus*, the main vector of Rocky Mountain spotted fever [48] and reported as a human-biting tick [49], was the most abundant and only tick collected, primarily infesting *C. hispidus* and *D. merriami*, which were also the most abundant rodents over the course of this research; it was also the second most prevalent parasite overall, and is the most abundant tick reported in the area [50]. This tick has been reported before in the area as the only tick infesting rodents [14] and is also a dominant parasite for dogs in the region [48]. Given its role as a vector of several pathogens to both animals and humans, it is important to monitor its populations. As far as fleas are concerned, two species were identified, *M. altipecten* and *M. dipodomys*; these have been reported to parasitize heteromyids in the area [14]; however, here, we report higher prevalences and abundance. *Meringis* spp. fleas have previously been associated with Bartonella [51,52], an important zoonotic bacterium. Research in these species as vectors of important pathogens is limited, and additional studies will be necessary to determine their role in pathogen transmission. Finally, only four lice, *Fahrenholzia* spp., were captured, all of them on a single individual *D. merriami*. This genus of louse is typically found in rodents like pocket mice and kangaroo rats [53], and their role in pathogen transmission is not well explored. These species of ectoparasites have already been described by other authors in areas close to the study area [14,51,54].

Previous studies have shown that wildlife serves as a reservoir of various zoonoses in geographical areas close to the present study [55,56] and that environmental factors and human behavior have favored changes in the disease cycle involving other peridomestic animals [57]. This research identified the etiological agent of plague (*Y. pestis*) in 4.85%, while rickettsiosis was found in 3.88% of the heteromyids sampled. These findings are consistent with previous research, as *Y. pestis* has been identified in rodent fleas in the state of Chihuahua [51], prairie dogs in New Mexico [58,59], and other wild mammals [56], with a high prevalence of plague antibodies [60].

On the other hand, the etiologic agent of tularemia was not identified (*F. tularensis*). Nevertheless, this bacterium has been reported in New Mexico and Arizona, USA [61], and Sonora, Mexico [62]. The absence of *F. tularemia* in this study does not imply its absence in the area, and this result could be influenced by factors such as sample size or the time of capture. Further studies should be considered in the future to calculate prevalence, including in domestic animals. Efforts to identify vectors and vector-borne diseases in both domestic and wild animals should be continued. There are reports of these zoonotic diseases in distant countries, which show, once again, that the problem is worldwide [46,63,64,65,66,67].

Findings of these significant zoonoses and their vectors underscore the need to understand the factors that drive parasitism. Previous studies have explored the relationship between parasitism and factors like body size [68], vegetation [69], and host sex [70]. Our results suggest that individuals with better body condition have a higher probability of pathogen infection. Although pathogens are often detrimental to host health, some studies suggest that animals in better body condition may better tolerate both parasites and pathogens; individuals with a higher parasite prevalence and parasite diversity show better body condition [71], suggesting that better body condition may facilitate infection control. Furthermore, some parasites seem to prefer hosts with better body condition because hosts with poor body condition do not provide enough resources [72]. Social mechanisms may also mediate the relationship between body condition and parasitism; dominant animals, with access to more and higher-quality resources [73,74], often engage in more social activity and increased activity, which can increase their exposure to parasites [75] and their risk of pathogen exposure [76]. Interestingly, non-infested individuals also presented a higher probability of pathogen infection; avoidance behaviors such as changes in social behavior and avoidance and removal of parasites [77] might account for this result. Furthermore, grooming behavior among kangaroo rats serves as a flea removal mechanism [78], and there are other mechanisms such as burrow cleaning [79] that already infected individuals recur to control the infestation even if they are already infected by the pathogen. Future studies could expand on localities; explore additional pathogens such as *Anaplasma* and *Ehrlichia*, which have also been reported in the area; and include rodent displacement and social behavior as a measure of pathogen infection risk.

## 5. Conclusions

We report *Rhipicephalus sanguineus*, *Meringis altipecten*, *M. dipodomys*, and *Fahrenholzia* spp. infesting seven heteromyid rodents at three locations of the Chihuahuan desert. Our statistical models suggest that *M. dipodomys* and *M. altipecten* may be adapted to infect a broad range of heteromyid rodents, and although these parasites have not been documented as primary vectors of important pathogens, additional research is necessary to determine their role in pathogen transmission. We further identified *Y. pestis* and *Rickettsia* spp. as important zoonotic agents. We found that non-parasite-infested individuals with better body condition have a higher probability of pathogen infection. Furthermore, we observed that blood samples had a higher probability of detecting pathogen-infected individuals, as compared to spleen or liver samples. Our results offer important insights into host–pathogen interactions and the role of body condition in pathogen status.

## Figures and Tables

**Table 2:** Number of each rodent species captured in the three study areas during the two sampling seasons in the northern Chihuahuan desert.

Rodent Species	First Season			Second Season			Total
	Las Palmas	Villa Luz	Ley Seis de Enero	Las Palmas	Villa Luz	Ley Seis de Enero	
*D. merriami*	31	9	13	26	14	13	106 (49.76%)
*D. ordii*	1	5	-	1	1	-	8 (3.75%)
*D. spectabilis*	1	-	-	1	-	1	3 (1.18%)
*Ch. hispidus*	1	12	-	11	2	21	47 (22.06%)
*Ch. eremicus*	-	-	4	2	6	15	27 (12.67%)
*P. flavus*	1	9	-	9	-	-	19 (8.92%)
*P. flavescens*	1	2	-	-	-	-	3 (1.40%)
Total	90			123			213 (100%)

**Table 3:** Prevalence (%), obtained from infested individuals/captured individuals of each species during both seasons. In parentheses in each parasite column is the number of collected ectoparasites.

Rodent Species	Prevalence (Infested Animals)	*R. Sanguineus*	*M. Altipecten*	*M. Dipodomys*	*Fahrenholzia* spp.
*D. merriami*	39.6% (42/106)	12% (48)	27% (62)	16% (32)	2% (4)
*D. ordii*	37.5% (3/8)	-	38% (3)	25% (3)	-
*D. spectabilis*	33.3% (1/3)	-	33% (4)	33% (2)	-
*C. hispidus*	48.9% (23/47)	28% (52)	4% (2)	2% (2)	-
*C. eremicus*	40.7% (11/27)	19% (15)	-	7% (1)	-
*P. flavus*	47.3% (9/19)	26% (19)	11% (2)	16% (3)	-
*P. flavescens*	0% (0/3)	-	-	33% (1)	-
Total	42% (89/213)	16.9% (134)	17.3% (73)	12.6% (43)	0.9% (4)

**Table 4:** GLM results analyzing the effects of explanatory variables over pathogen infection in heteromyid rodents at an influential zone of the APFF Médanos de Samalayuca.

	Contrast	Estimate	Z-Value	Res. Dev	*p*	AUC
Intercept		−2.11				0.78
Sample	blood:spleen	0.25	0.37	86.84	*	
	blood:liver	17.24	0.01			
Infested	Inf:non-inf	−0.91		84.58		

AUC: area under the curve, from the ROC (receiver operating characteristic). *p* < 0.05 = *. AIC saturated model = 106.7, AIC selected model = 92.58.

**Table 5:** Estimates of Beta model coefficients to assess the influence of ectoparasites and rodent species on infection prevalence.

	Variable	Estimate	Std. Error	Z-Value	*p*-Value
Intercept	*Fahrehnholzia* spp.:*C. eremicus*	−3.39	0.55	−6.146	***
Ectoparasite	*M_altipecten*	1.28	0.45	2.844	**
	*M_dipodomys*	1.54	0.44	3.469	***
	*R_sanguineus*	0.76	0.46	1.636	0.10
Rodent	*C_hispidus*	0.08	0.59	0.143	0.89
	*D_merriami*	0.87	0.55	1.585	0.11
	*D_ordii*	0.70	0.56	1.262	0.21
	*D_spectabilis*	0.75	0.56	1.353	0.18
	*P_flavescens*	0.07	0.59	0.111	0.91
	*P_flavus*	0.71	0.56	1.273	0.20

Significance values are indicated by asterisks: *** *p* < 0.001, ** *p* < 0.01. pseudo-R^2^ = 0.4439. Phi value = 11.05.

## Data Availability

Data are available from the authors upon reasonable request.
